# *Streptococcus parasuis*, an Emerging Zoonotic Pathogen, Possesses the Capacity to Induce Cerebral Inflammatory Responses

**DOI:** 10.3390/pathogens12040600

**Published:** 2023-04-15

**Authors:** Kexin Qi, Xueli Yi, Mingliu Wang, Jianping Wang, Hui Sun, Pujun Liang, Jianguo Xu, Han Zheng

**Affiliations:** 1 State Key Laboratory of Infectious Disease Prevention and Control, National Institute for Communicable Disease Control and Prevention, Chinese Center for Disease Control and Prevention, Beijing 102206, China; 2 Center for Clinical Laboratory Diagnosis and Research, The Affiliated Hospital of Youjiang Medical University for Nationalities, Baise 533000, China; 3 Guangxi Zhuang Autonomous Region Center for Disease Prevention and Control, Nanning 530021, China; 4 Nanxishan Hospital of Guangxi Zhuang Autonomous Region, Guilin 541002, China

**Keywords:** *S. parasuis*, cerebral inflammation, histopathological changes, proinflammatory mediators, TLRs, astrocyte, microglia, phagocytosis

## Abstract

To date, three *Streptococcus parasuis* strains, BS26, BS27, and NN1, have been isolated from the blood cultures of patients with peritonitis, pneumonia, and arthritis, indicating that *S. parasuis* is an emerging threat to susceptible people. There is thus an urgent need to further evaluate the pathogenesis of *S. parasuis* clinical strains in order to design efficient anti-inflammatory strategies. Our previous study demonstrated the capacity of *S. parasuis* clinical strains to enter the central nervous system (CNS) of infected mice. However, the characteristics and inflammatory mechanism of CNS infections caused by *S. parasuis* are still non-available. In the present study, we investigated the proportion and time of two clinical *S. parasuis* strains NN1 and BS26 infected mice that developed neurological symptoms. The characteristics of histopathological changes and the cerebral immune response in mice with neurological symptoms were analyzed. Furthermore, we evaluated the roles of microglia and astrocytes in the *S. parasuis* clinical strain-induced cerebral inflammation. Our data indicated that *S. parasuis* clinical strains possess a high potential to induce cerebral inflammation in susceptible people at the early phase of infection. Our study contributes to increasing the understanding of the pathogenicity of *S. parasuis* and the inflammatory mechanisms of the brain against infection caused by *S. parasuis*.

## 1. Introduction

*Streptococcus suis* is an important zoonotic pathogen that primarily causes meningitis, sepsis, endocarditis, arthritis, and pneumonia in pigs and humans [1]. Recently, *S. suis* serotypes 20, 22, and 26 were reclassified as *Streptococcus parasuis* [2]. Although most *S. parasuis* strains have been isolated from the saliva of clinically healthy pigs, the presence of *S. parasuis* in diseased pigs and calves with pneumonia or systemic infection (meningitis, arthritis, endocarditis, or septicemia) indicates that this species may be pathogenic in pigs and calves [3,4,5,6]. In our previous study, two *S. parasuis* clinical strains, BS26 and BS27, were isolated from a patient with peritonitis and a patient with pneumonia and arthritis, respectively [7]. In the present study, a third *S. parasuis* clinical strain, NN1, was isolated from a patient with pneumonia. This indicates that the zoonotic pathogen *S. parasuis* is an emerging threat to public health and underscores the urgent need to further evaluate the pathogenesis of *S. parasuis* clinical strains. In our previous study, the capacity of *S. parasuis* clinical strains to cause systemic infections and histopathological lesions in lungs and livers of infected C57BL/6 mice were revealed [7]. However, the mechanism of immunity response against the infection caused by *S. parasuis* is still poorly understood. An important hallmark of the pathogenicity of *S. suis* is its ability to cross the blood–brain barrier (BBB) and induce an inflammatory response in the brain, which leads to damage to the central nervous system (CNS) [8]. Our previous study demonstrated the capacity of *S. parasuis* clinical strains to enter the CNS of infected mice [7]. In the present study, we investigated the characteristics and mechanism of cerebral inflammation against the infection caused by *S. parasuis* clinical strains. Firstly, we evaluated the proportion and time of the neurological symptoms developed in mice infected with two S. parasuis clinical strains. In addition, the characteristics of histopathological changes and the cerebral immune response in mice with neurological symptoms were analyzed. Furthermore, we explored the roles of microglia and astrocytes in the *S. parasuis* strain-induced cerebral inflammation.

## 2. Materials and Methods

### 2.1. Case Description, Sequencing, and Phylogenetic Analysis of S. parasuis Strain NN1

The peripheral blood of a patient with pneumonia was collected, sterilized, and cultured on Columbia blood plates at 37 °C, 5% CO_2_ overnight in 2020. The pure cultured strain NN1 was obtained and confirmed as *S. parasuis* species by the amplification of a nearly complete 16S rRNA gene [9] and the *S. parasuis*-specific *recN* gene [10].

The host of the isolated NN1 strain was an 81-year-old female patient who was admitted to the First Affiliated Hospital of Guangxi Medical University in Nanning, China, on 10 October 2020 because of a pulmonary infection characterized by the acute onset of fever (body temperature of 38.6 °C), chills, chest distress, cough, and expectoration. Eight months earlier, the patient had been diagnosed with B-cell non-Hodgkin lymphoma. The patient had been using daily oral treatment to control her blood pressure for 17 years. The serum level of hypersensitive C-reactive protein (hs-CRP) (measured by immunoturbidimetry, c16000, Abbott, North Chicago, IL, USA) and total white blood cell (WBC) count (measured by flow cytometry, c16000, Abbott) were 110.9 mg/L and 3.34 × 10^9^/L, respectively. A computed tomography (CT) image indicated inflammation in the lower lobes of both lungs. Meropenem and vancomycin sodium pentahydrate were administered as antibiotic therapy. The patient was discharged when her symptoms were relieved.

The complete genome of *S. parasuis* strain NN1 was obtained using the PacBio Sequel platform and Illumina NovaSeq PE150. The sequencing libraries were constructed, and the sequence was analyzed according to the methods described in our previous study [7].

In our previous core genome phylogenetic analysis, 13 *S. parasuis* genomes were clustered into three discrete lineages [7]. *S. parasuis* clinical strains BS26 and BS27 showed a phylogenetic affinity with *S. parasuis* strain 4253, and the three strains were clustered into lineage 1. In the present study, the lineage 1 representative genomes of BS26, BS27, and 4253, the lineage 2 representative genome of SUT-286, and the lineage 3 representative genome of SUT-7 were selected. For comparison, the genomes of NN1, SUT-503 (accession no. AP024280), and H35 (accession no. CP076721) were included in the phylogenetic analysis. *S. suis* type-strain NCTC10234T (accession no. LS483418) was used as the outgroup to root the tree. The phylogenetic tree based on 353 core genes of nine genomes was constructed according to the method described in our previous study [7].

Distributions of 129 putative *S. suis* virulence-related genes [11] among the *S. parasuis* strains were investigated. Genes having a global match region at <80% of the nucleotide acid sequence with an identity of <80% were determined as absent.

### 2.2. The Antimicrobial Susceptibility Profile of S. parasuis Strain NN1

Antimicrobial susceptibility testing was performed by assessing the minimum inhibitory concentration (MIC) of NN1 using MIC test strips (Liofilchem, Roseto degli Abruzzi, Italy). The MIC test strips contained a gradient of concentrations of penicillin G (0.002–2 μg/mL), cefaclor (0.016–256 μg/mL), tetracycline (0.016–256 μg/mL), erythromycin (0.016–256 μg/mL), azithromycin (0.016–256 μg/mL), clindamycin (0.016–256 μg/mL), streptomycin (0.064–1024 μg/mL), kanamycin (0.016–256 μg/mL), spectinomycin (0.064–1024 μg/mL), gentamicin (0.016–256 μg/mL), and trimethoprim–sulfamethoxazole (0.002–32 μg/mL). *S. pneumoniae* ATCC 49619 was used for quality control. The breakpoints recommended by the 2016 Clinical and Laboratory Standard Institute (CLSI) guidelines (M100-S26) for *Streptococcus* spp. (*Viridans* group) and *Streptococcus pneumoniae* were used for penicillin G, cefaclor, tetracycline, azithromycin, erythromycin, clindamycin, and trimethoprim–sulfamethoxazole. No breakpoint values were available for streptomycin, kanamycin, gentamicin, or spectinomycin for *Streptococci*, so their breakpoints were obtained from previous studies [12,13].

### 2.3. Cell Isolation and Culture

Primary astrocytes were obtained from the brains of nine neonatal (3-day-old) C57BL/6 mice for each experiment. The primary astrocytes were cultured and purified as described in our previous study [14]. BV2 cells were purchased from the Cell Resource Center of the Institute of Basic Medical Sciences (IBMS) at the Peking Union Medical College (CAMS/PUMC) in Beijing, China. This cell line exhibits the morphology and functional characteristics of microglia and is a valid substitute for primary microglial cells [15].

The glial cells were cultured in Dulbecco’s modified eagle medium (DMEM) (Hyclone, Beijing, China) supplemented with 10% heat-inactivated fetal bovine serum (FBS, Invitrogen, Grand Island, NY, USA). The glial cells were detached with 0.25% trypsine–0.53 mM EDTA. The cell suspensions were mixed thoroughly and counted by Neubauer-improved counting chamber (Assistent, Bavarian, Germany). For each experiment, 1 mL of cells/well was plated on 24 well-plates at a density of 3 × 10^5^ cells/mL and maintained in 5% CO_2_ at 37 °C for 48 h to allow cell confluence (approximately 1 × 10^6^ cells/mL) before the infection assays. The medium was changed every 24 h.

### 2.4. Experimental Infection

In our previous study, the virulence levels of *S. parasuis* clinical strains BS26 and BS27 were similar [7]. *S. parasuis* clinical strains BS26 and NN1 were selected in the present study. For comparison, the highly pathogenic *S. suis* strain P1/7 (serotype 2 and sequence type 1) isolated from a pig with fatal meningitis [16] was included as a control. The strains were grown overnight on Columbia blood agar base plates (Oxoid, London, UK) under 5% CO_2_ at 37 °C, and five isolated colonies per strain were inoculated into 5 mL of Todd–Hewitt broth (THB; Oxoid) without shaking under 5% CO_2_ at 37 °C until the OD600 value reached 0.6, which corresponded to 1 × 10^9^ CFU/mL, 8 × 10^8^ CFU/mL, and 5 × 10^8^ CFU/mL for P1/7, BS26, and NN1, respectively. The strains were washed twice in phosphate-buffered saline (PBS) (pH 7.4; Gibco) before infection. Serial dilutions of the bacterial suspension were plated onto THB agar plates to determine the final CFU/mL for each experiment.

#### 2.4.1. Survival Assay

The survival rate of mice infected with *S. parasuis* strain NN1 was compared with those of mice infected with *S. suis* strain P1/7 and *S. parasuis* strain BS26. For the survival assay, C57BL/6 mice (6 weeks old, female) were injected intraperitoneally with 5 × 10^7^ CFUs of the different strains in 1 mL THB (or 1 mL THB only for the control). Each infection group contained ten mice, and the mock-infected group contained five mice. The mortality was recorded every 6 h for the first 24 h post-infection and every 12 h from 24 h to 72 h post-infection. The experiment was performed independently in duplicate. Survival rates were calculated by the Kaplan–Meier method, and the mean survival rates were presented in survival curves.

#### 2.4.2. Histopathology and RNA Extraction of the Brains of Mice with Neurological Symptoms

To minimize the mortality of infected mice, C57BL/6 mice (6 weeks old, female) were injected intraperitoneally with 2 × 10^7^ CFUs of the different strains in 1 mL THB. In our previous study, the duration of *S. parasuis* clinical strains BS26 and BS27 present in the brain of C57BL/6 mice injected intraperitoneally with 5 × 10^7^ CFUs was 72 h post-infection [7]. In the present study, the time points of experimental infection in vivo were designed to be 12 h, 24 h, 48 h, and 72 h post-infection. A total of 120 infected and 12 non-infected mice were included in the experiment. The infection groups each contained 40 mice and were divided into four subgroups, with 10 mice for each designed time point. The control group contained three non-infected mice for each time point.

The mortality and neurological symptoms were observed every 12 h from 0 h to 72 h post-infection. At each designated time, the survival mice in each infection group were euthanized by cervical dislocation, and the brains of mice with neurological symptoms were collected aseptically. Half of the brains were fixed for 24 h at room temperature in 4% buffered formalin. After paraffin embedding, conventionally dewaxed tissue sections were stained with hematoxylin and eosin (H&E) [17]. The images were acquired and analyzed by light microscopy. The total RNA of the other half of the brains was extracted by the RNeasy Plus Universal Mini Kit (Qiagen) to analyze the transcription of TLRs and proinflammatory mediator genes.

#### 2.4.3. Interaction with Primary Astrocytes and BV2 Cells

Prepared 1 × 10^6^ CFU/mL bacterial suspensions in DMEM with high glucose medium (1 mL per well, 3 wells per strain for each time point) were added to primary astrocytes and BV2 cells (multiplicity of infection [MOI] 1:1), respectively. The plates were centrifuged at 500× *g* for 10 min and incubated under 5% CO_2_ at 37 °C for 2, 4, 8, 12, 18, and 24 h. The supernatants obtained at 8, 12, 18, and 24 h post-infection were collected to measure the levels of TNF-α, IL-6, and MCP-1 by ELISA (R&D Systems, Minneapolis, MN, USA). The cytokine values were expressed as mean + standard deviation. The remaining cell monolayers were washed with a cell culture medium and lysed to obtain the total cellular RNA using TRIzol (Invitrogen) following the protocol. In addition, Pam3CSK4 (a TLR1/2 agonist), FSL-1 (a TLR2/6 agonist), and ODN2395 (a TLR9 agonist) (InvivoGen, San Diego, CA, USA) were added as positive controls at concentrations of 0.5 µg/mL or 10 µg/mL (for ODN2395). Each experiment was repeated three times.

#### 2.4.4. Phagocytosis Assays

The strains were either nonopsonized or serum-opsonized. For these assays, 500 µL of each strain (2 × 10^6^ CFU/mL) was incubated for 30 min at 37 °C with 20% normal C57BL/6 mouse serum, 20% heat-inactivated (56 °C, 30 min) mouse serum, and PBS, respectively. The strains were then collected by centrifugation at 12,000× *g* for 10 min and resuspended in an antibiotic-free medium. Strains incubated in the presence of serum showed a twofold increase in CFUs. Thus, to normalize the infectious dose to that of PBS-treated strains, opsonized strains were twofold diluted before the test. Finally, BV2 cells interacted with 1 × 10^6^ CFUs of nonopsonized, serum-opsonized, and heat-inactivated serum-opsonized strains (MOI 1:1), respectively. At 90 min post-infection, cell monolayers were washed twice with warm culture media and re-incubated for 1 h with a medium containing penicillin G (5 μg/mL) and gentamicin (100 μg/mL). After antibiotic treatment, the supernatant was plated onto THB agar plates to confirm that the antibiotics effectively killed the extracellular bacteria. Subsequently, cell monolayers were washed three times and lysed with 1 mL of sterile cold water. After vigorous pipetting to ensure complete cell lysis, viable intracellular bacteria were counted by plating serial dilutions of the lysates onto THB agar. Each experiment was repeated three times, and the number of CFUs recovered per well (mean + standard deviation) was determined.

### 2.5. Transcription Analysis of TLRs and Proinflammatory Mediators by Real-Time Reverse Transcription Quantitative PCR

The concentration and purity of all RNA samples obtained from in vitro and in vivo infection experiments were measured with a Qubit 4.0 Fluorometer (Thermo Fisher Scientific, Loughborough, UK). Next, 20 ng of total RNA was used to directly amplify various genes of TLRs and proinflammatory mediators with the HiScript^®^ II One Step qRT-PCR SYBR Green Kit (Vazyme biotech, Nanjing, China). The PCR amplification protocol for all RNA samples was 15 min at 50 °C, followed by 30 s at 95 °C and 40 cycles of denaturing for 10 s at 95 °C and 30 s at 60 °C. A melting curve step (15 s at 95 °C followed by 60 s at 60 °C and 15 s at 95 °C) was added at the end of each amplification. Each 20 μL reaction contained forward and reverse primers at a concentration of 200 nM. The primers of the TLRs and proinflammatory mediators were listed in Table 1. The *gapdh* gene (encoding glyceraldehyde 3-phosphate dehydrogenase) was used as a normalizing gene. Non-infected primary astrocytes, BV2 cells, and mice were used as the calibration reference in the corresponding analyses, respectively. The quantitation of the differences between groups was calculated using the 2^−ΔΔCt^ method. The transcription values were expressed as mean + standard deviation.

### 2.6. Statistics

The survival rates of different groups were compared by the log-rank test. Statistical analyses of the cytokine data and transcription data were performed by Student’s unpaired *t*-test and ANOVA test. Differences between groups were considered significant at *p* < 0.05.

### 2.7. Ethical Approval

This study and the application of the animal experiments (code 2022-003) were reviewed and approved by the ethics committee of the National Institute for Communicable Disease Control and Prevention, Chinese Center for Disease Control and Prevention.

### 2.8. Nucleotide Sequence Accession Numbers

The complete genome sequence of *S. parasuis* strain NN1 was deposited in GenBank under the accession number CP073632.

## 3. Results

### 3.1. Evaluation of the Genomic Characteristics, Antimicrobial Profile, and Virulence Genes of S. parasuis Strain NN1

*S. parasuis* strain NN1 showed 100% 16S rRNA gene sequence similarity with strains BS26 and BS27. In addition, the phylogenetic tree based on the alignment of core genes of eight *S. parasuis* genomes and one *S. suis* genome indicated that NN1 had a phylogenetic affinity with the other two clinical strains BS26 and BS27 (Figure S1). Strains 4253 and H35 were clustered into the same lineage with clinical strains. The phylogenetic analysis based on the gene presence and absence of 14 *S. parasuis* strains also clustered strains 4253 and H35 into the same clade with two clinical strains BS26 and BS27 [18]. The *cps* gene cluster of NN1 was clustered into type X and shared 99.2% sequence similarity with BS26 [7].

NN1 was resistant to tetracycline, erythromycin, and azithromycin, with MIC values of 16 μg/mL, 32 μg/mL, and >256 μg/mL, respectively. The tetracycline resistance gene *tet*(M) was present in NN1, whereas genes encoding tetracycline resistance were absent in BS26 and BS27. Three *S. parasuis* clinical strains harbored *msr*(D) and *mef*(A) genes encoding macrolides resistance. The presence of two copies of *msr*(D) and *mef*(A) in NN1 may partially explain its higher resistance to erythromycin and azithromycin in comparison with BS26 and BS27.

NN1 was susceptible to penicillin G, cefaclor, clindamycin, streptomycin, kanamycin, spectinomycin, gentamicin, vancomycin, rifampicin, chloramphenicol, and trimethoprim–sulfamethoxazole.

The presence of 129 putative virulence genes of *S. suis* were investigated in three *S. parasuis* clinical strains. No significant difference in the distribution of putative virulence genes was observed among *S. parasuis* clinical strains. Indeed, 47 of them were present in all three *S. parasuis* clinical strains. In addition, the *oppa* gene encoding the ABC transporter substrate-binding protein was only present in *S. parasuis* clinical strains BS26 and BS27, whereases the *hhly3* gene encoding amino acid transporter protein was only present in *S. parasuis* clinical strain NN1 (Supplemental Table S1).

### 3.2. Survival Rates of Mice Infected with S. suis Strain P1/7, S. parasuis Strain BS26, and S. parasuis Strain NN1 Differed

The survival level of mice infected with NN1 was significantly higher than those of mice infected with BS26 and *S. suis* strain P1/7. Mice infected 5 × 10^7^ CFUs of NN1 had a 90% survival rate at 24 h post-infection, whereas the survival rates of mice infected with *S. suis* strain P1/7 and *S. parasuis* strain BS26 were 10% and 30% at the same time point, respectively. At 72 h post-infection, the survival rate of mice infected with NN1 (75%) was significantly higher than those of mice infected with *S. suis* strain P1/7 (10%) and *S. parasuis* strain BS26 (20%) (Figure S2).

### 3.3. Two S. parasuis Strains Induced Neurological Symptoms in Infected Mice from 24 h Post-Infection

To reduce the mortality of the infected mice and obtain enough observations, the infection dose decreased to 2 × 10^7^ CFUs for each strain. In the present study, 100% (40/40), 77% (22/30), 45% (9/20), and 40% (4/10) of mice infected with *S. parasuis* strain BS26 survived at 12 h, 24 h, 48 h, and 72 h post-infection, respectively. The survival rates of mice infected with NN1 at 12 h, 24 h, 48 h, and 72 h post-infection were 100% (40/40), 100% (30/30), 80% (16/20), and 60% (6/10), respectively. Compared with the two *S. parasuis* strains, *S. suis* strain P1/7 possessed a higher capacity to induce lethal infection at 24 h post-infection; the survival rates at 12 h, 24 h, 48 h, and 72 h post-infection were 83% (33/40), 27% (8/30), 20% (4/20), and 20% (2/10), respectively.

The survival mice infected with BS26 and NN1 displayed neurological symptoms from 24 h post-infection. The appearance time and proportion of neurological symptoms in survival mice differed between mice infected with BS26 and NN1. Among the mice infected with BS26, half (20/40) of the survival mice displayed ataxia of mainly paresis of the hindlimb (median time 24 h), and 30% (6/20) of the survival mice with paresis of the hindlimb also displayed bending of the head toward one side and walking in circles as the infection progressed (median time 60 h). Among the mice infected with NN1, 45% (18/40) of the survival mice displayed ataxia of mainly paresis of the hindlimb (median time 48 h), and over 20% (4/18) of the survival mice with paresis of the hindlimb also displayed bending of the head toward one side and walking in circles as the infection progressed (median time 72 h). However, we could not rule out the possibility that the mice sacrificed at earlier time points would have developed neurological symptoms at later time points; thus, the proportion of survival mice with neurological symptoms may be underestimated.

Notably, no neurological symptoms were observed in survival mice infected with *S. suis* strain P1/7 before 72 h post-infection. This result was in agreement with a previous study, which reported the appearance of clinical symptoms of meningitis in *S. suis* ST1-infected mice from four days post-infection [19].

### 3.4. Investigation of Histopathological Changes in the Brains of Survival Mice with Neurological Symptoms

**Figure 1:**
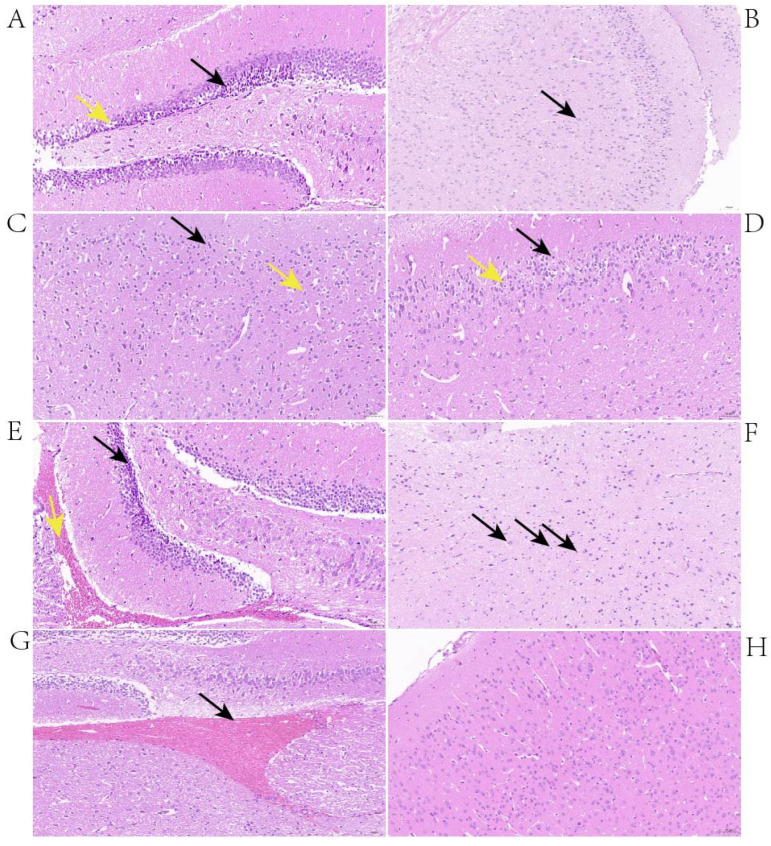
Histopathological changes in the brains of 2 × 10^7^ CFUs of *S. parasuis* strains BS26 and NN1 which infected mice with neurologic symptoms. (**A**) Micrograph of brain from *S. parasuis* strain BS26infected a mouse at 24 h post-infection. Neuronal deformation and vacuolization of the neuronal cytoplasm (yellow arrowhead) and neuronal atrophy (black arrowhead) were shown. (**B**) Micrograph of brain from *S. parasuis* strain BS26 infected a mouse at 24 h post-infection. Engulfment of neuronal debris by microglia (black arrowhead) was shown. (**C**) Micrograph of brain from *S. parasuis* strain NN1 which infected a mouse at 24 h post-infection. Vacuolization of the neuronal cytoplasm (yellow arrowhead) and neuronal atrophy (black arrowhead) were shown. (**D**) Micrograph of brain from *S. parasuis* strain NN1 infected mouse at 24 h post-infection. Microgliosis (yellow arrowhead) and neuronal atrophy (black arrowhead) were shown. (**E**) Micrograph of brain from *S. parasuis* strain NN1 which infected a mouse at 48 h post-infection. Hemorrhage (yellow arrowhead) and neuronal atrophy (black arrowhead) were shown. (**F**) Micrograph of brain from *S. parasuis* strain BS26 which infected a mouse at 72 h post-infection. Slight neutrophil infiltration (black arrowhead) was shown. (**G**) Micrograph of brain from *S. parasuis* strain NN1 which infected a mouse at 72 h post-infection. Hemorrhage (black arrowhead) was shown. (**H**) Micrograph of brain from mock-infected mouse at 72 h post-infection. H&E staining, ×200 magnification, scale: 50 μm.

The histopathological changes in the brains of mice with neurological symptoms were investigated. At 24 h post-infection, the changes observed in the brains of mice infected with BS26 were neuronal atrophy, neuronal deformation, and vacuolization of the neuronal cytoplasm in the hippocampus (Figure 1A). In addition, the brains of BS26-infected mice exhibited the engulfment of neuronal debris by microglia from 24 h post-infection (Figure 1B). At the same time point, the histopathological changes in mice infected with NN1 appeared in the cerebral cortex and included neuronal deformation, vacuolization of the neuronal cytoplasm, and microgliosis (Figure 1C,D). At 48 h post-infection, the aforementioned neuronal atrophy, neuronal deformation, and vacuolization of the neuronal cytoplasm appeared in both the hippocampus and the cerebral cortex of mice infected with BS26. In contrast, at the same time point, the significant histopathological changes which appeared in the brains of NN1-infected mice were hemorrhagic foci (Figure 1E). At 72 h post-infection, the significant histopathological changes in the brains of BS26-infected mice with neurological symptoms were slight neutrophil infiltration (Figure 1F). In contrast, hemorrhagic foci had appeared in the brains of almost all NN1-infected mice with neurological symptoms at the same time point (Figure 1G).

### 3.5. Both S. parasuis Strains Induced Rapid Upregulation of Proinflammatory Mediators and TLR Genes Transcription in the Brains of Mice with Neurological Symptoms

**Figure 2:**
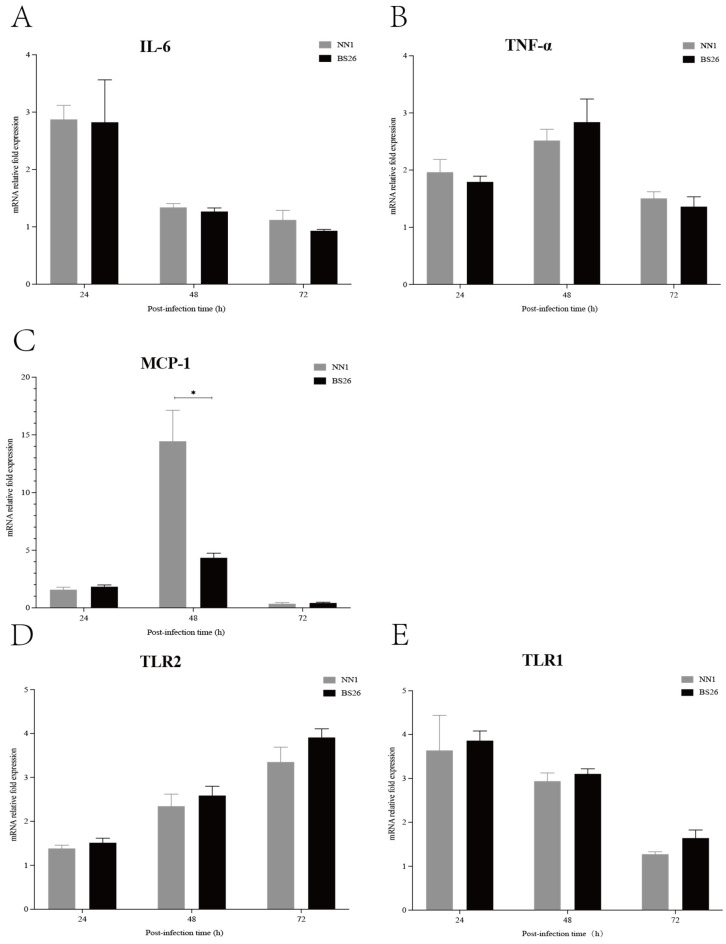
The transcription levels of IL-6 (**A**), TNF-α (**B**), MCP-1 (**C**), TLR2 (**D**), and TLR1 (**E**) genes in 2 × 10^7^ CFUs of *S. parasuis* strains BS26 and NN1 infected mice with nervous signs at 24 h, 48 h, and 72 h post-infection. The transcription levels were calculated after normalizing cycle thresholds against the “housekeeping” gene *gapdh* using the 2^−ΔΔCt^ method. All data were presented as mean + standard deviation. Statistical analyses of the data were performed using the Student’s unpaired *t* test. *: significantly different (*p* < 0.05) between the groups.

The transcription levels of IL-6, TNF-α, and MCP-1 genes in the brains of mice with neurological symptoms were investigated. The transcription levels of the IL-6 gene in the brains of mice infected with both *S. parasuis* strains were slightly upregulated at 24 h post-infection and then gradually returned to the baseline level at 48 h post-infection (Figure 2A). The transcription patterns of the TNF-α and MCP-1 genes were similar in mice infected with the two *S. parasuis* strains that showed neurological symptoms, which slightly upregulated at 24 h post-infection, peaked at 48 h post-infection and then gradually returned to the baseline level at 72 h post-infection (Figure 2B,C). No significant differences in transcription levels of the IL-6 and TNF-α genes were observed between mice infected with BS26 and NN1 throughout the experiment (Figure 2A,B), whereas a significantly higher transcription level of the MCP-1 gene was observed in NN1-infected mice at 48 h post-infection (Figure 2C).

TLRs play a pivotal role in the innate immune response by recognizing the pathogen-associated molecular patterns (PAMPs) of invading pathogens. In the present study, we investigated the kinetics of TLR1, TLR2, TLR6, and TLR9 gene transcription in the brains of infected mice with neurological symptoms. TLR2 gene transcription patterns in the brains of BS26- and NN1-infected mice with neurological symptoms were upregulated at 48 h post-infection and peaked at 72 h post-infection (Figure 2D). Notably, TLR1 gene transcription was also upregulated in the brains of BS26- and NN1-infected mice with neurological symptoms, but a different transcription pattern in which transcription peaked at 24 h post-infection and then gradually decreased to the baseline level at 72 h post-infection was observed (Figure 2E). The transcription levels of the TLR1 and TLR2 genes in the brains of BS26- and NN1-infected mice with neurological symptoms were no different (Figure 2D,E).

### 3.6. The Capacity of S. parasuis Strains to Induce Proinflammatory Mediators Production by Primary Astrocytes and BV2 Cells Was Higher Than That of S. suis Strain P1/7 at the Early Phase of Interaction

Activated astrocytes and microglia played critical roles in the CNS inflammatory response against *S. suis* strains infection by producing proinflammatory mediators [14,20,21]. In this study, the capacity of *S. parasuis* strains to induce proinflammatory cytokine and chemokine production by primary astrocytes and BV2 cells was investigated and compared with that of *S. suis* strain P1/7.

**Figure 3:**
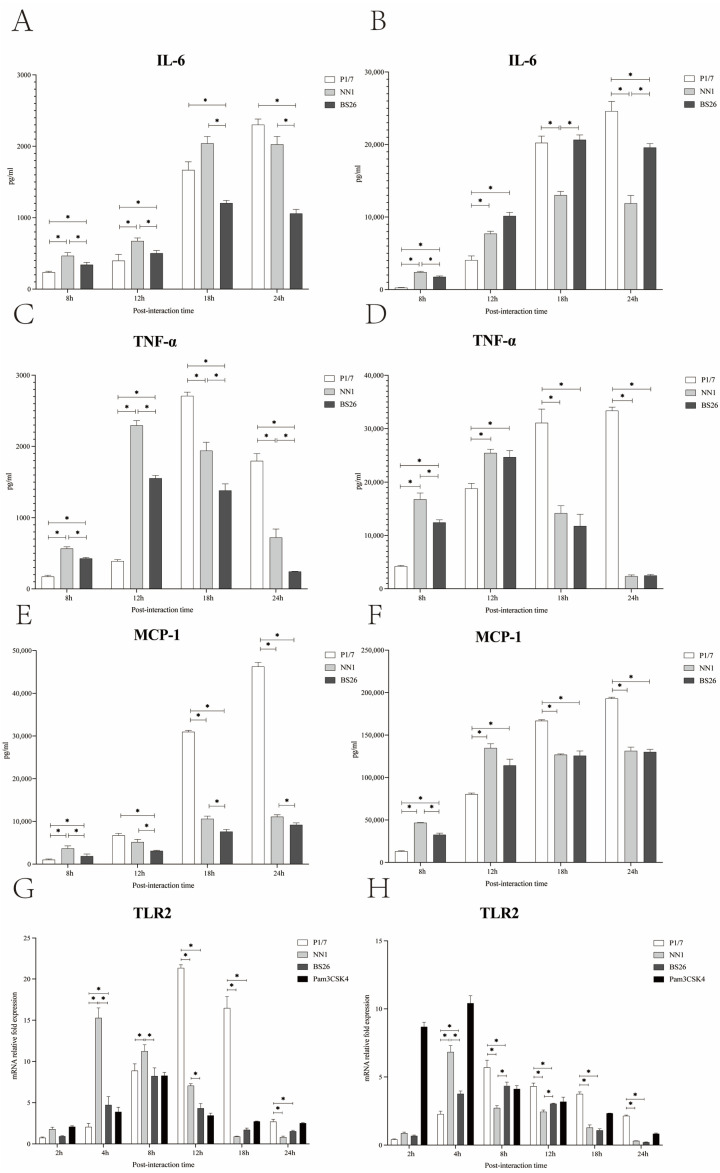
The levels of IL-6 by primary astrocytes (**A**) and BV2 cells (**B**), TNF-α by primary astrocytes (**C**) and BV2 cells (**D**), MCP-1 by primary astrocytes (**E**) and BV2 cells (**F**), and TLR2 gene transcription by primary astrocytes (**G**) and BV2 cells (**H**) interacted with 1 × 10^6^ CFUs of *S. parasuis* strain BS26, *S. parasuis* strain NN1, and *S. suis* strain P1/7, respectively. Glial cells stimulated with either Pam3CSK4 or FSL-1 were used as positive control, which are specific ligands for TLR2/1 and TLR2/6, respectively. All data were presented as mean + standard deviation of three independent experiments. Statistical analyses of the data were performed using the ANOVA test. *: significantly different (*p* < 0.05) between the groups.

The levels of IL-6 in both primary astrocytes and BV2 cells infected with the two *S. parasuis* strains peaked at 18 h post-infection and then gradually decreased at 24 h post-infection (Figure 3A,B). The levels of TNF-α in both primary astrocytes and BV2 cells infected with the two *S. parasuis* strains peaked at 12 h post-infection and gradually decreased thereafter (Figure 3C,D). Of the two *S. parasuis* strains, NN1 possessed a higher capacity to induce IL-6, TNF-α, and MCP-1 production by primary astrocytes throughout the experiment, and by BV2 cells at 8 h post-infection, whereas BS26 induced higher IL-6 production by BV2 cells from 18 h post-infection (Figure 3A–F).

The differences between *S. suis* strain P1/7 and the two *S. parasuis* strains in the kinetics of the proinflammatory mediators secreted by primary astrocytes and BV2 cells were observed. At 8 h and 12 h post-infection, the levels of TNF-α, IL-6, and MCP-1 in both primary astrocytes and BV2 cells infected with the two *S. parasuis* strains were significantly higher than those of cells infected with *S. suis* strain P1/7, except for the MCP-1 in primary astrocytes at 12 h post-infection (Figure 3A–F). As the infection progressed, TNF-α and MCP-1 levels in both primary astrocytes and BV2 cells infected with *S. suis* strain P1/7 were significantly higher than those of cells infected with the two *S. parasuis* strains at 18 h and 24 h post-infection (Figure 3C–F). The levels of IL-6 in both primary astrocytes and BV2 cells infected with *S. suis* strain P1/7 peaked at 24 h post-infection (Figure 3A,B). At 24 h post-infection, *S. suis* strain P1/7 induced a significantly higher level of IL-6 production by BV2 cells than the two *S. parasuis* strains (Figure 3B).

### 3.7. The Activation of Primary Astrocytes and BV2 Cells by S. parasuis Strains Was Mediated by TLR2

In this study, the kinetics of the TLR1, 2, 6, and 9 genes transcription in primary astrocytes and BV2 cells infected with the two *S. parasuis* strains were investigated and compared with those of corresponding glial cells infected with *S. suis* strain P1/7. The TLR2 gene transcription levels in primary astrocytes and BV2 cells infected with NN1 peaked at 4 h post-infection, whereas the TLR2 gene transcription levels in glial cells infected with BS26 peaked at 8 h post-infection (Figure 3G,H). Of the two *S. parasuis* strains, NN1 induced higher transcription levels of the TLR2 gene in primary astrocytes before 18 h post-infection and in BV2 cells at 4 h post-infection, whereas BS26 induced higher transcription levels of the TLR2 gene in BV2 cells at 8 h and 12 h post-infection (Figure 3G,H).

At 4 h post-infection, the transcription levels of the TLR2 gene in both primary astrocytes and BV2 cells infected with two *S. parasuis* strains were significantly higher than those of cells infected with *S. suis* strain P1/7; conversely, *S. suis* strain P1/7 induced the highest transcription levels of the TLR2 gene among three strains in primary astrocytes and BV2 cells from 12 h and 8 h post-infection, respectively (Figure 3G,H).

Neither *S. suis* strain P1/7 nor the two *S. parasuis* strains upregulated the transcription levels of the TLR1, 6, and 9 genes in both glial cells.

### 3.8. The Capacity of S. parasuis Strains to Resist Phagocytosis by BV2 Cells Was Lower Than That of S. suis Strain P1/7 under Various Conditions

**Figure 4:**
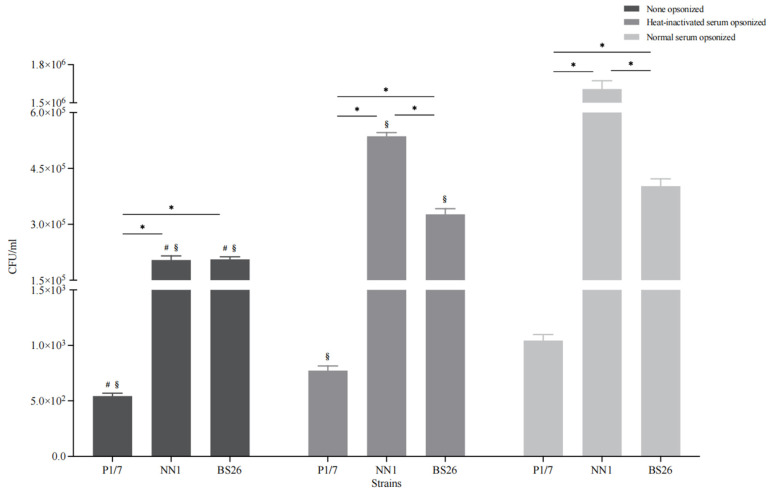
Phagocytosis levels of *S. parasuis* strain BS26, *S. parasuis* strain NN1, and *S. suis* strain P1/7 under non-opsonic or opsonic conditions. Strains were either non-opsonized or pre-opsonized with 20% normal mouse serum or heat-inactivated mouse serum for 30 min prior to interaction with BV2 cells for 90 min. All data were presented as mean + standard deviation of three independent experiments. Statistical analyses of the data were performed using the Student’s unpaired *t* test. *: significantly different (*p* < 0.05) between the groups. §: significantly different (*p* < 0.05) compared to the corresponding strain pre-opsonized with normal mouse serum. #: significantly different (*p* < 0.05) compared to the corresponding strain pre-opsonized with heat-inactivated mouse serum.

Microglia are phagocytes that play critical roles in the removal of invading pathogens [22]. Under non-opsonic conditions, the phagocytosis levels of the two *S. parasuis* strains (more than 10% of the initial inoculum for both strains) were significantly higher than that of *S. suis* strain P1/7 (less than 0.1% of the initial inoculum) (Figure 4). In addition, the capacity of BS26 to resist phagocytosis by BV2 cells was significantly higher than that of NN1 (Figure 4).

To evaluate the contribution of the complement pathway to the phagocytosis of *S. suis* strain P1/7 and two *S. parasuis* strains, bacterial opsonization with normal and heat-inactivated mouse serum was performed prior to the experiment, respectively. Normal serum pre-opsonization resulted in an eightfold increase in the internalization of *S. parasuis* strain NN1, whereas the internalization of *S. suis* strain P1/7 and *S. parasuis* strain BS26 increased by twofold (Figure 4). Notably, the phagocytosis levels of the three heat-inactivated serum pre-opsonized strains were significantly lower than those of corresponding normal serum pre-opsonized strains, although they were significantly higher than those of corresponding non-opsonized strains (Figure 4).

## 4. Discussion

To date, three strains of *S. parasuis*, an emerging zoonotic pathogen, have been isolated from the blood cultures of patients with peritonitis, pneumonia, and arthritis. The putative *S. suis* virulence-related genes present in *S. parasuis* clinical strains were widespread in intermediately and lowly pathogenic *S. suis* strains [23]. Interestingly, none of the *S. parasuis* genomes harbored “classical” *S. suis* virulence markers *mrp*, *sly,* and *epf*. Virulence genes preferentially present in highly pathogenic and epidemic *S. suis* strain, such as *ofs*, *sao*, *nisK*, *nisR*, *salK*, *salR*, *revS*, *virB4*, *virD4*, and *SSU05_0473* genes [23,24,25], were absent in three *S. parasuis* clinical strains. It indicated that the pathogenic mechanism of *S. parasuis* clinical strains may differ from that of highly pathogenic *S. suis* strains. The survival assay revealed substantial differences in virulence levels of the three *S. parasuis* clinical strains, even though they exhibited phylogenetic affinity and a nearly identical distribution of putative virulence genes.

A better understanding of the pathogenesis of *S. parasuis* is important to establish effective anti-inflammatory strategies. Hitherto, information on the pathogenicity of *S. parasuis* and the mechanisms underlying the host inflammatory response initiated by the *S. parasuis* infection is still scarce. *S. suis* has a phylogenetic affinity with *S. parasuis*. Meningitis is the most common clinical syndrome of *S. suis* infection in humans [26]. Evaluation on the potential of *S. parasuis* to initiate inflammation in CNS is critically needed. The presence and replication in the CNS are the first steps for pathogens to establish infection. Our previous study demonstrated the capacity of *S. parasuis* strains to enter the CNS of infected mice [7]. However, the cerebral immune response following *S. parasuis* infection and its subsequent damage to the CNS tissue remain unclear. In the present study, we investigated the features of the cerebral inflammatory response against *S. parasuis* infection in survival mice with neurological symptoms and the roles of resident glial cells in the inflammation.

The two *S. parasuis* strains induced neurological symptoms in infected C57BL/6 mice from 24 h post-infection. The major neurological symptoms of *S. parasuis*-infected mice were paresis of the hindlimb, bending of the head toward one side, and walking in circles. It is noteworthy that a higher proportion and more rapid appearance of neurological symptoms were observed in survival mice infected with *S. parasuis* strain BS26 than those of survival mice infected with *S. parasuis* strain NN1. Compared with survival mice infected with *S. parasuis* strains, no neurological symptoms were observed in survival mice infected with *S. suis* strain P1/7. This may be partially attributable to the high mortality of mice infected with *S. suis* strain P1/7 from 12 h post-infection.

Various histopathological changes were observed in the brains of mice with neurological symptoms infected with the two *S. parasuis* strains. At the early phase of infection, the common histopathological changes of mice infected with both *S. parasuis* strains were neuronal atrophy, neuronal deformation, and vacuolization of the neuronal cytoplasm. However, these histopathological changes were mainly present in the cerebral cortex of mice infected with *S. parasuis* strain NN1, whereas they appeared in both the hippocampus and cerebral cortex of mice infected with *S. parasuis* strain BS26. From 24 h post-infection, microgliosis in the cerebral cortex and the engulfment of neuronal debris by microglia were observed in the brains of mice infected with NN1 and BS26, respectively. At the later phase of infection, a higher proportion of hemorrhage and slight neutrophil infiltration was present in the brains of NN1- and BS26-infected mice, respectively. These findings indicate that the two *S. parasuis* strains induced different histopathological changes in the CNS, and the related pathogenesis of these changes may differ between the two strains.

The rapid and effective initiation of the host immune response is critical for the CNS to clear invading pathogens, but it also results in neurological dysfunction by producing high levels of proinflammatory cytokines and chemokines [27]. In the present study, the two *S. parasuis* strains sequentially induced the upregulation of IL-6, TNF-α, and MCP-1 gene transcription in the brains of mice with neurological symptoms from 24 h post-infection. The significantly higher CSF concentrations of IL-6, TNF-α, and MCP-1 are usually found in the acute phase of patients with bacterial meningitis, especially in patients with a fatal outcome [28,29]. This indicates that proinflammatory mediators may play critical roles in the histopathological changes and related neurological symptoms induced by *S. parasuis* strains.

The upregulation of IL-6 gene transcription was more rapid than that of the TNF-α and MCP-1 genes in the brains of both *S. parasuis* strains infected mice with neurological symptoms. This indicates that IL-6, an important inducer of acute phase proteins, may play a key role in the initiation and amplification of the inflammatory response against invading *S. parasuis* strains. *S. parasuis* strain NN1 induced a significantly higher transcription level of the MCP-1 gene than *S. parasuis* strain BS26 did at 48 h post-infection, whereas no differences in the transcription levels of the TNF-α and IL-6 genes were observed between the two *S. parasuis* strains. MCP-1 is responsible for the recruitment of neutrophils and mononuclear cells from the peripheral circulation into the inflammation site of the CNS [30]. However, no obvious neutrophil infiltration in the brains of *S. parasuis* strain NN1-infected mice with neurological symptoms was observed. This may be partially attributable to the drastic decline in the MCP-1 transcription level after reaching the peak.

TLRs play a critical role in inflammation by activating downstream signaling pathways, leading to the expression of a variety of proinflammation cytokines and chemokines [31]. TLR2 activation was involved in the pathogenesis of meningitis induced by *S. suis* strains [14,19,21]. TLR1 and TLR6 binded with TLR2 to form receptor clusters (heterodimers) in response to different G^+^ bacterial components [32]. In the present study, the upregulation of TLR1 and TLR2 gene transcription was observed in the brains of two *S. parasuis* strains-infected mice with neurological symptoms. *S. parasuis* strains induced a rapid upregulation of TLR1 gene transcription, whereas they induced a comparatively delayed upregulation of TLR2 gene transcription. The delayed expression of TLR2 was also observed in CD1 mice infected with *S. suis* ST1 strain [19]. It is possible that TLR1 played an important role in the initiation of cerebral inflammation induced by the two *S. parasuis* strains. Although only TLR1 and TLR2 gene transcription was upregulated, we cannot exclude the possibility that other receptors were involved in the pathogenesis of cerebral inflammation induced by *S. parasuis* strains. Further research is needed to investigate the immune pathways exploited by *S. parasuis* strains to induce the production of various proinflammation cytokines and chemokines in the CNS.

Resident glial cells play critical roles in the inflammatory response following bacterial invasion of the brain. Previous studies have shown that primary astrocytes and BV2 cells played key roles in the CNS inflammation triggered by *S. suis* strains [14,20,21]. In the present study, the two *S. parasuis* strains induced higher levels of proinflammatory cytokine and chemokine production by primary astrocytes and BV2 cells compared with *S. suis* strain P1/7 at the early phase of interaction. This may partially lead to the high proportion of survival mice infected with two *S. parasuis* strains developing neurological symptoms at the early phase of infection. In contrast, *S. suis* strain P1/7 induced significantly higher levels of proinflammatory cytokine and chemokine production by primary astrocytes and BV2 cells at the later phase of interaction. No clinical symptoms of meningitis were observed in P1/7-infected mice at the early phase of infection, which may be partially attributed to the delayed production of proinflammatory mediators by astrocytes and microglia.

NN1 possessed a higher capacity to induce proinflammatory cytokine and chemokine production by primary astrocytes throughout the experiment, whereas BS26 induced higher levels of IL-6 production by BV2 cells at the later phase of interaction. We propose that activated astrocytes may play a critical role in the NN1-induced cerebral inflammatory response, whereas the role of microglia may be more crucial in the BS26-induced CNS inflammation.

The rapid production of proinflammatory mediators by astrocytes and BV2 cells which interacted with the two *S. parasuis* strains may be partially due to the significant upregulation of TLR2 gene transcription at the very early phase of interaction. Moreover, the transcription levels of the TLR2 gene in both primary astrocytes and BV2 cells which interacted with the two *S. parasuis* strains were significantly higher than those of cells that interacted with *S. suis* strain P1/7 at the very early phase of interaction. These results underline the importance of TLR2 in mediating *S. parasuis*-induced cerebral inflammation. Neither *S. parasuis* strains upregulated TLR1 gene transcription in primary astrocytes or BV2 cells. It is possible that endothelial and ependymal cells, rather than resident microglia and astrocytes, were the main cellular sources of upregulated TLR1 gene transcription in the CNS.

The transience of proinflammatory mediator’s transcription upregulation in the brains of two *S. parasuis* strains infected mice with neurological symptoms may be partially due to their low capacity to resist serum-mediated opsonic phagocytosis. Conversely, *S. suis* strain P1/7 possessed a significantly higher capacity to resist serum-mediated opsonic phagocytosis than the two *S. parasuis* strains did. The bacterial burden and duration in the brains of mice infected with *S. suis* strain P1/7 were significantly greater than those of mice infected with the *S. parasuis* strains [7]. The combination of the higher capacity to activate glial cells at the later phase of infection and higher bacterial burden and duration in the brain led to a prolonged and stronger immune response in the brains of mice infected with *S. suis* strain P1/7. This is supported by the findings of a previous study, in which severe symptoms of meningitis and histopathological lesions were observed in CD1 mice infected with *S. suis* strain ST1 from 4 days to 9 days post-infection [19].

The complement system is an important element of the innate immune defense against many bacterial pathogens. Our results indicated that the loss of complement activity resulted in a significant reduction in the internalization of *S. parasuis* strains. Furthermore, the observed differences in phagocytosis levels between heat-inactivated serum opsonic and non-opsonic conditions indicate that other heat-stable serum factors, such as albumin and fibronectin, are also involved in opsonic phagocytosis of *S. parasuis* strains. BS26 possessed a significantly higher capacity to resist serum-mediated opsonic phagocytosis than NN1. Kozel et al. proposed that complement opsonization and capsule size have an inverse relationship with phagocytosis: when the capsule size increases, the efficacy of complement-mediated phagocytosis decreases [33]. The *cps* gene cluster of *S. parasuis* strain NN1 shared a 99.2% sequence similarity with *S. parasuis* strain BS26. Further studies are needed to investigate the differences in CPS expression in vivo between the two *S. parasuis* strains.

In conclusion, *S. parasuis* clinical strains BS26 and NN1 induced transient and significant innate immunity response in the brain, mainly through the sequential activation of TLR1 and TLR2 at the early phase of infection, which then resulted in histopathological lesions and the appearance of neurological symptoms in mice. TLR2-mediated activation of astrocytes and microglia played important roles in the cerebral inflammatory response induced by BS26 and NN1, respectively. Our data suggest that *S. parasuis* clinical strains possess a high potential to induce cerebral inflammation in susceptible people at the early phase of infection. Further studies are needed to investigate the differences in the pathogenesis of inducing cerebral inflammation between *S. parasuis* strains BS26 and NN1.

## Figures and Tables

**Table 1:** The sequences of primers used in the real-time quantitative PCR.

Gene	Forward Primer	Reverse Primer	Amplicon Size
TLR1	CACAGCTCCTTGGTTTTAATG	TGGGTATAGGACGTTTCTGTAG	102 bp
TLR2	TGGAGCATCCGAATTGCATCACCG	GAGCGGCCATCACACACCCC	193 bp
TLR6	GCCTCCCTGGCTCCTGGCTA	AGGGACTTTGCTGAGTTTCTGATCCA	139 bp
TLR9	CCGTCAGTGCTGGAAATAG	CGATGGGTTTTCTGTCTTGG	108 bp
IL-6	CTTCCATCCAGTTGCCTTCT	CTCCGACTTGTGAAGTGGTATAG	139 bp
TNF-α	TTGTCTACTCCCAGGTTCTCT	GAGGTTGACTTTCTCCTGGTATG	109 bp
MCP-1	CTCACCTGCTGCTACTCATTC	ACTACAGCTTCTTTGGGACAC	102 bp
GADPH	CCCGTAGACAAAATGGTGAAG	GACTGTGCCGTTGAATTTG	186 bp

## Data Availability

The data presented in this study are openly available in the article and its Supplementary Materials.

## Supplementary Materials

The following supporting information can be downloaded at: https://www.mdpi.com/article/10.3390/pathogens12040600/s1, Figure S1: Core-genome phylogeny of nine *Streptococcus* genomes in the present study; Figure S2: Survival curves of mice injected with 5 × 10^7^ CFU of live *S. parasuis* strains BS26 and NN1, *S. suis* strain P1/7, and THB only as control group. Table S1: The presence of 129 putative virulence genes of *S. suis* in the genomes of three *S. parasuis* clinical strains.
