# Supplementary material for: Streptococcus parasuis, an Emerging Zoonotic Pathogen, Possesses the Capacity to Induce Cerebral Inflammatory Responses
Source: Pathogens. 2023 Apr 15;12(4):600. doi: 10.3390/pathogens12040600 (PMC10141694; doi:10.3390/pathogens12040600)
Supplement: Supplementary file 1 [file pathogens-12-00600-s001.zip › Supplemental Figure S2.pdf]

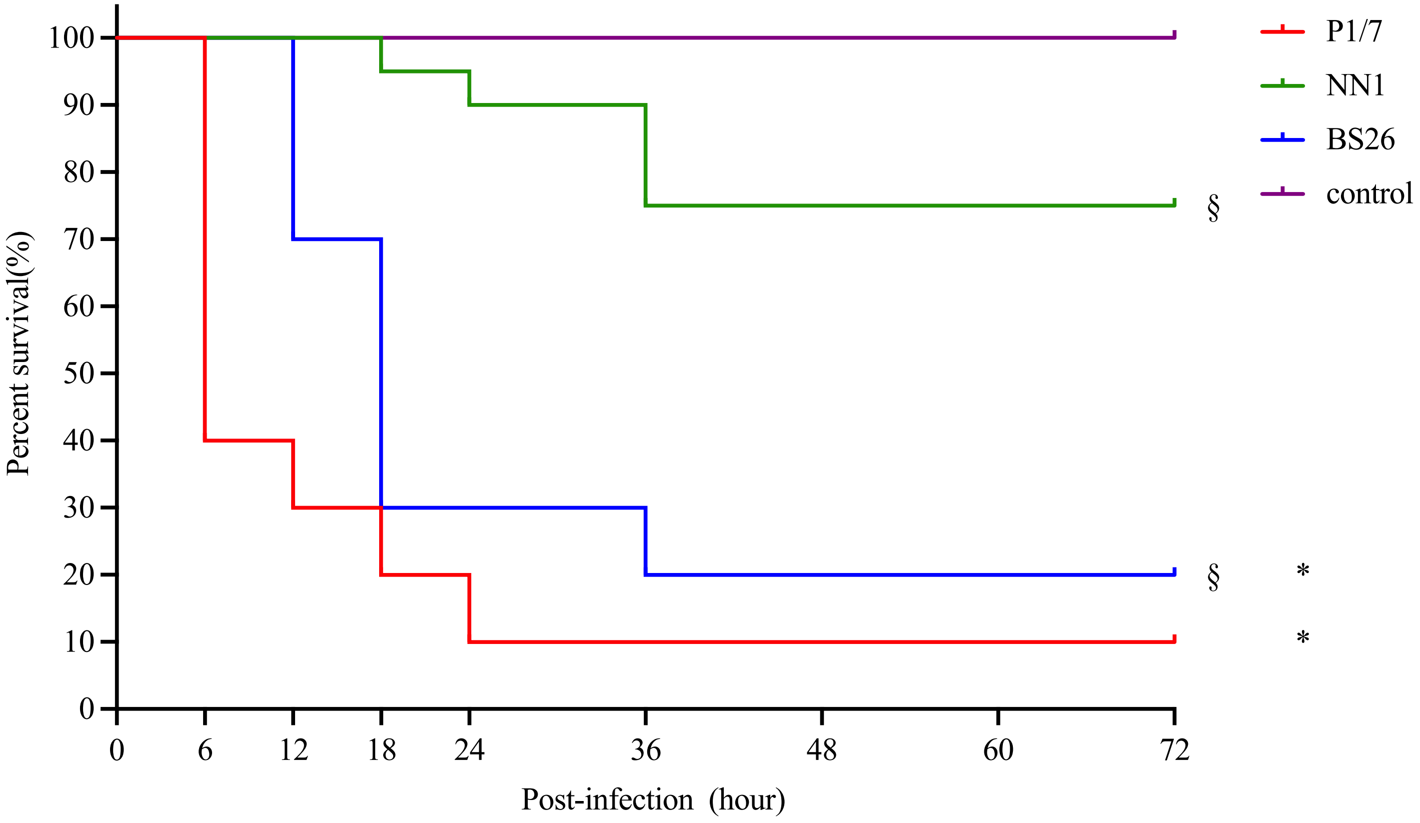

Figure S2. Survival curves of mice injected with 5 x 107 CFU of live *S. parasuis* strains BS26 and NN1, *S. suis* strain P1/7, and THB only as control group.
