# Supplementary material for: Streptococcus parasuis, an Emerging Zoonotic Pathogen, Possesses the Capacity to Induce Cerebral Inflammatory Responses
Source: Pathogens. 2023 Apr 15;12(4):600. doi: 10.3390/pathogens12040600 (PMC10141694; doi:10.3390/pathogens12040600)
Supplement: Supplementary file 1 [file pathogens-12-00600-s001.zip › Supplementary Table S1.pdf]

Table S1. The presence of 129 putative virulence genes of *S. suis* in the genomes of three *S. parasuis* clinical strains.

	BS26	BS27	NN1
purD	+	+	+
05SSU0053			
adcR	+	+	+
impdh2	+	+	+
EF-G	+	+	+
sspep			
pgk	+	+	+
glnA	+	+	+
ef			
Hp197			
dppIV			
copper-exporting ATPase	+	+	+
treR	+	+	+
HP0245			
glutamate dehydrogenase	+	+	+
Hp272			
adhE	+	+	+
38 kDa-1	+	+	+
38 kDa-2			
hrcA	+	+	+
grpE	+	+	+
perR			
Tig	+	+	+
103/adhesion protein			
htpS			
fba	+	+	+
codY	+	+	+
luxS			
stp			
stk			
vrasr			
BgaC			
tran			
SSU05_0473			
srtF			
collagenase			
peptidase	+	+	+
EF-Tu	+	+	+
pk	+	+	+
ides			
permease			
gntR			
neuB			

neuC			
Flps			
arginine deminase system			
arcH			
argR			
dltA			
05SSU0660			
mrp			
sspA			
clpX	+	+	+
guaA	+	+	+
SpyM3-0908			
nisK			
nisR			
hhly3			+
sp1			
salR			
salK			
traG			
Ssads			
IgA1			
relQ			
rex			
srtA			
HP1083	+	+	+
cdd	+	+	+
ciaH			
ciaR			
pept	+	+	+
prsa			
pepf	+	+	+
autolysin			
SSU05_1311			
covR			
sao			
ccpA	+	+	+
sly			
feoB			
fbp	+	+	+
eno	+	+	+
HP1538			
clpP	+	+	+
gtfA	+	+	+
lgt	+	+	+
pgdA			
pgm	+	+	+
ihk			
irr			

ofs			
yzpa			
dpr	+	+	+
ham1			
HP1717			
mannose-specific EIIAB: Phosphotransferase	+	+	+
ManM	+	+	+
ManN	+	+	+
ManO			
SerS	+	+	+
virA			
kar	+	+	+
IgdE			
scrB	+	+	+
scrA	+	+	+
oppa	+	+	
1910HR			
1910HK			
gp92			
endo D			
purA	+	+	+
SsnA			
mutT	+	+	+
pnuc			
nadR	+	+	+
sbp2			
rgg			
ApuA	+	+	+
troA			
revS			
relA	+	+	+
sntA			
sbp1			
pili			
collagen-binding protein			
gdpp			
gidA	+	+	+
guaB	+	+	+
