# Supplementary figures and images for: Streptococcus parasuis, an Emerging Zoonotic Pathogen, Possesses the Capacity to Induce Cerebral Inflammatory Responses
Source: Pathogens. 2023 Apr 15;12(4):600. doi: 10.3390/pathogens12040600 (PMC10141694; doi:10.3390/pathogens12040600)

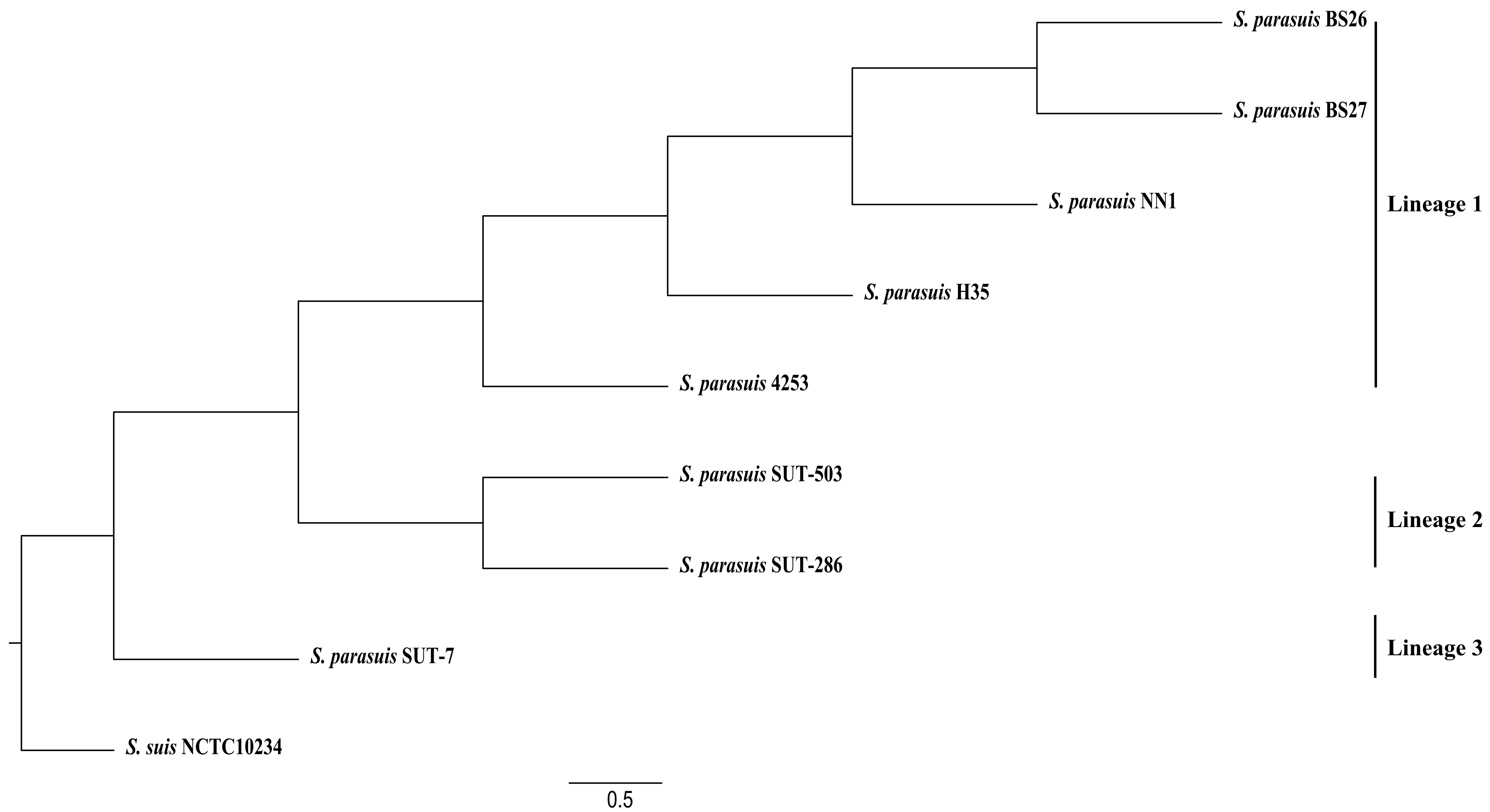

Figure S1. Core-genome phylogeny of nine Streptococcus genomes in the present study.

Supplement: Supplementary file 1 [file pathogens-12-00600-s001.zip › Supplemental Figure S1.pdf]
